# Establishing A Sustainable Low-Cost Air Quality Monitoring Setup: A Survey of the State-of-the-Art

**DOI:** 10.3390/s22010394

**Published:** 2022-01-05

**Authors:** Mannam Veera Narayana, Devendra Jalihal, S. M. Shiva Nagendra

**Affiliations:** 1 Electrical Engineering, Indian Institute of Technology, Madras 600036, India; dj@ee.iitm.ac.in; 2 Civil Engineering, Indian Institute of Technology, Madras 600036, India; snagendra@iitm.ac.in

**Keywords:** air quality monitoring, low-cost sensors, calibration, evaluation metrics, citizen science applications

## Abstract

Low-cost sensors (LCS) are becoming popular for air quality monitoring (AQM). They promise high spatial and temporal resolutions at low-cost. In addition, citizen science applications such as personal exposure monitoring can be implemented effortlessly. However, the reliability of the data is questionable due to various error sources involved in the LCS measurement. Furthermore, sensor performance drift over time is another issue. Hence, the adoption of LCS by regulatory agencies is still evolving. Several studies have been conducted to improve the performance of low-cost sensors. This article summarizes the existing studies on the state-of-the-art of LCS for AQM. We conceptualize a step by step procedure to establish a sustainable AQM setup with LCS that can produce reliable data. The selection of sensors, calibration and evaluation, hardware setup, evaluation metrics and inferences, and end user-specific applications are various stages in the LCS-based AQM setup we propose. We present a critical analysis at every step of the AQM setup to obtain reliable data from the low-cost measurement. Finally, we conclude this study with future scope to improve the availability of air quality data.

## 1. Introduction

Air pollution is a global challenge. Rapid growth in industrialization and urbanization are associated with growing air pollution [1,2]. Scientific studies have shown that the excessive presence of air pollutants, such as oxides of nitrogen (NO_X_), oxides of sulphur (SO_X_), particulate matter (PM), carbon monoxide (CO) and Ozone (O_3_), can cause severe health problems ranging from breathing issues to mortality [3,4,5]. A study on 29 Indian cities with a population of more than 1 million estimated 114,700 deaths in 2016 due to PM_2.5_ (particles of size ≤2.5 μm) exposure alone. The same study estimated mean deaths of 20,300 in Delhi, one of India’s highly polluted cities [6]. Air pollution also leads to several environmental issues such as ozone layer depletion, acid rains, global warming, reduction of plant growth and crop yield, and the deterioration of building structures [7,8,9]. In developing countries, the problem is becoming more acute day by day [10]. Intensive urban air quality management plans (UAQMP) are being developed and implemented worldwide at various scales (national, state/city, local) to tackle alarming air pollution levels [10].

High air pollution levels characterise many cities in India, and many times their ambient pollution levels exceed the national ambient air quality standards (NAAQS). According to a WHO (World Health Organization) report, fourteen out of the fifteen most polluted cities are in India [11]. New Delhi is one of the most polluted cities globally, and during the winter season, the mist and fog formed due to higher air pollution levels cause visibility problems in the city. For instance, flights often get cancelled in and out of New Delhi airport due to the reduced visibility associated with air pollution. The main sources of ambient air pollution in India are residential and commercial biomass burning, coal-burning for energy generation, industrial emissions, agricultural stubble burning, waste burning, construction activities, brick kilns, transport vehicles, and diesel generators [12]. Further, it becomes more severe due to overpopulation and uncontrolled urbanisation and industrialisation development. Social disparities and lack of information intensify the problem further.

In addition, we can find spatial heterogeneity in India. We can witness diverse climatological conditions between southern India and northern India. Northern India is landlocked, while seas surround southern India. The main reason for the cold climate of the north Indian areas may be the landlocked geography. Due to these climate conditions, pollutants become trapped in the lower atmosphere and create more severe problems. In southern India, we can experience a tropical climate.

Evidence of the adverse effects of air pollution on health has grown in India. According to a report by the Indian council of medical research (ICMR), one in eight deaths in India are caused by air pollution. Studies from India have shown that short-term and long-term exposure is associated with disease burden and mortality [13]

At present, in India, air pollution is monitored with traditional monitoring methods with fixed stations, and they are expensive, sparsely distributed and require high maintenance. Air pollution is a complex phenomenon that shows high spatial and temporal variations. At industrial and high traffic areas, it changes spatially within meters and over time within hours. For example, Menon et al., reported a change in particulate concentration at high business streets during the morning, afternoon and evening for a tropical coastal city in India [14]. Citizen science applications such as personal exposure monitoring and occupational exposure monitoring need high spatial and temporal resolutions [15,16]. Air quality monitoring (AQM) at a finer and a more granular scale helps to deploy better air pollution management and mitigation plans.

Despite high pollution levels and high mortality rates, the country has a minimal number of AQM stations, far less than required [17]. One main reason for maintaining fewer air quality stations is high-cost involvement. To overcome the limitations in the traditional methods, a relatively new paradigm involving low cost sensors (LCS) has been proposed by researchers. Recent well-funded projects listed by Morawaska et al. [18] and Chojer et al. [19] indicate a paradigm shift of air pollution measurement with LCS replacing the conventional devices. Implementing low-cost air quality monitoring methods in developing countries like India has become a relevant solution for deploying a nationwide air quality monitoring network that helps to create a nationwide dataset on air pollution required to raise pollution awareness. LCS devices can bridge gaps between sparse government measurements and high spatial-temporal air quality data requirements.

LCS are popular for affordability, compactness, low power consumption and capturing high spatial and temporal variations. With the advancement of technology, sensors are available for measuring a number of pollutants. Sensor boxes/nodes/motes are constructed by integrating LCS with microcontroller and additional components (Global positioning system (GPS), Global System for Mobile communication (GSM) etc.). Real-time affordable multi-pollutant monitor (RAMP) [20], AirU pollution monitor [21], Particulate monitor devices (Atmos) [22], ARISense [23] and captor nodes [24] are examples of such sensor boxes/nodes constructed for air quality measurement. Several researchers assessed the feasibility of air pollution measurement with LCS in long-term deployments with larger area coverage. They recommended the state-of-the-art-low cost sensing with regular calibrations [25,26,27,28]. At the same time, it is reported that there was a drop in accuracy when LCS were shifted from laboratory to field due to environmental effects (humidity and temperature) and the presence of other pollutants [29,30,31,32]. Hence the LCS data are less accurate than desirable. Though there are extensive studies, only a few studies achieved the standards of US EPA (US environmental protection agency) or EEA (European Environment Agency) for a short duration. Due to the data reliability questions, regulatory bodies have been slow in adopting LCS for AQM.

In order to establish a sustainable AQM setup with LCS that produces reliable data for a longer duration, we need to consider several issues that influence the LCS performance. This study consolidates all these issues reported in the literature and proposes a framework to establish a reliable AQM setup with LCS that helps to improve the air quality in India. We firmly believe the same can be applied to the rest of the world.

### 1.1. Literature Review

This study is conducted based on the articles obtained through scientific databases of google scholar, IEEE explorer, Scopus, Web of Science and ACM digital library. The search has been done using the combination of keywords: low-cost sensors, air quality monitoring, calibration, applications, air quality setup. We considered articles that are available on or before 30 April 2021. We collected a total of 256 studies through the above mentioned searches. In the initial filtering, we eliminated duplicate articles obtained through different searches. The number of articles left after this filtering is 203.

We separated survey/review articles from other studies (total number of review articles obtained is 16). This separation helps us to achieve the present scenario of low-cost sensors in air quality monitoring and the gap that needs to be addressed. Various surveys we come across and their insights are listed in Table 1. From these review articles, we identified that different authors follow different procedures to establish and calibrate an LCS based AQM setup. Hence we recognize that there is a need for a structured procedure to establish a sustainable, low-cost AQM setup that produces reliable data for a longer duration. After that, we went through the abstracts of the rest of the studies and eliminated similar case studies of the same location to avoid redundancy (number of articles is five). At the same time, we pruned the articles unrelated to this study (number of articles is 39).

**Figure 1:**
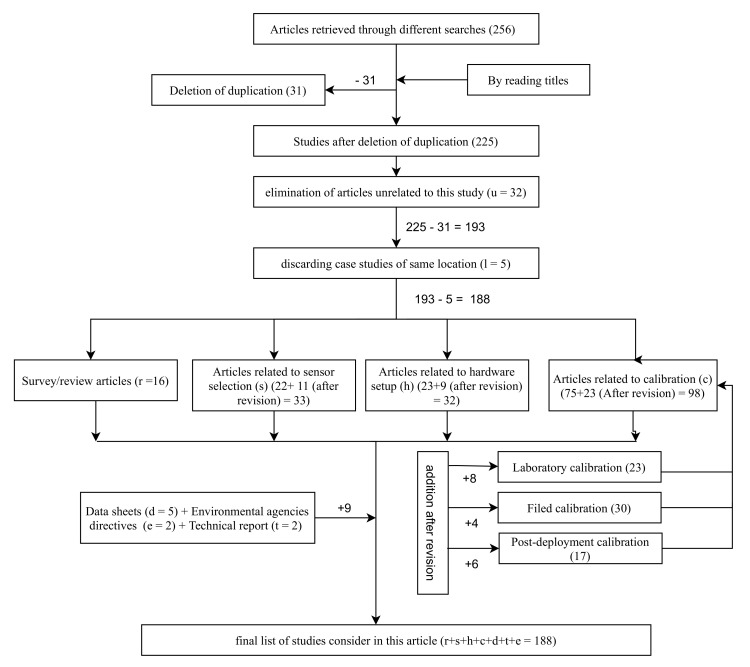
Flow chart explaining literature review methodology.

In addition, we reviewed the data sheets of various commercially available LCS (number of data sheets studied are five) that are searched with the keywords data sheet and sensor name in Google. Further, we consider studies related to various advanced communication techniques that are suitable for LCS sensor data transfer. The flow chart in Figure 1 illustrates the literature review methodology.

Note: After the first revision, we removed five identified references as entered twice in the references list. Hence the final list of articles in the Figure 1 is 183 (r + s + h + c + d + t + e = 188 − 5 = 183).

### 1.2. Our Contribution

We identified the need for a comprehensive report on the complete procedure to be followed in order to establish an air pollution monitoring set up with LCS. This study consolidates all methods and techniques followed by various researchers as a single framework. We provide critical analysis at every step in the framework. This study helps to establish a better AQM setup quickly and understand its performance. LCS can comply wit the regulatory bodies and it can make more air quality data available to citizens.

**Figure 2:**
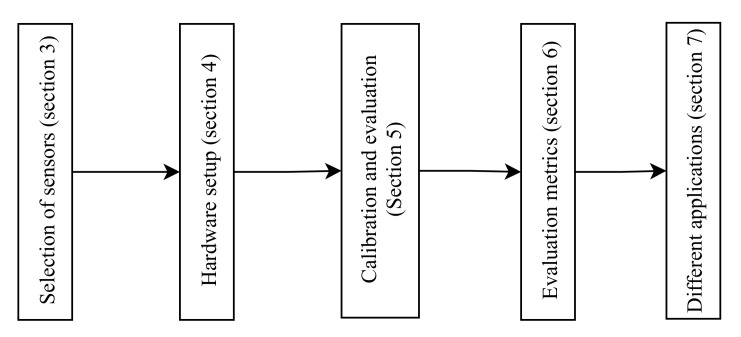
Flow chart illustrating the road-map of the study.

Road-map: Section 2 explains the overall framework briefly and, in the subsequent sections, we will discuss each step in detail. In Section 3, we illustrate how to select LCS for a particular pollutant. Hardware establishment procedure is available in Section 4. Section 5 deals with calibration and evaluation. Section 6 describes different metrics to understand the performance of low-cost sensors. Various applications of LCS are covered in Section 7. Conclusions & future scope are presented in Section 8. Figure 2 illustrates the flow of this study.

## 2. LCS Based Air Pollution Measurement

**Figure 3:**
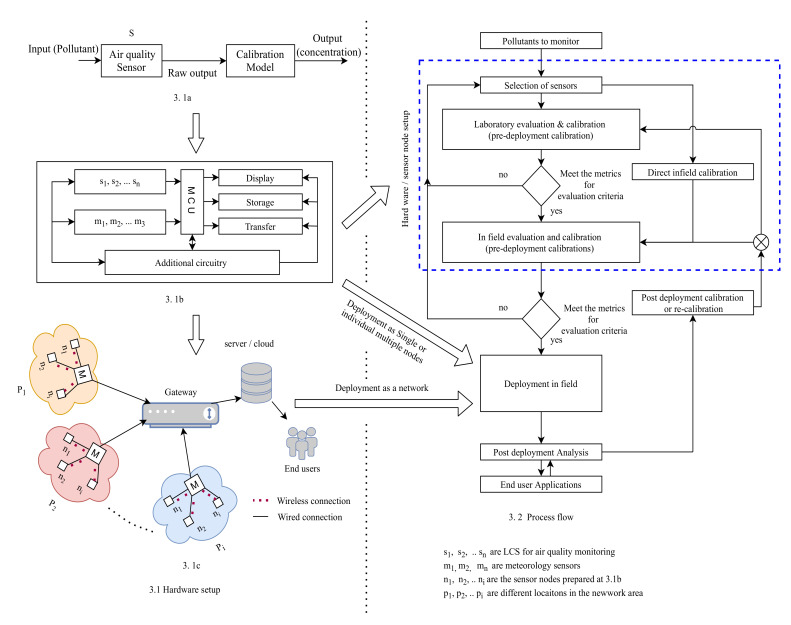
LCS based air pollution measurement framework. **3.1** Hardware setup; **3.1a** block box representation of LCS; **3.1b** Sensor node/box setup; **3.1c** typical sensors network; **3.2** process flow.

A consolidated framework for AQM with LCS is shown in Figure 3. The left of the Figure 3 (3.1) illustrates the hardware setup at different stages and, the right half (Figure 3 (3.2)) demonstrates the process flow. The hardware setup is discussed in detail in Section 4. The process flow (flowchart in 3.2) for AQM with LCS is explained in the following steps, and each step is discussed in detail in the subsequent sections.

Step 1: Select appropriate sensors for the given set of conditions and applications (Section 3).
Step 2: Calibrate the selected sensors in a laboratory under controlled environmental conditions at different concentrations of pollutants (Section 5.1.1). Once the laboratory calibration is finished, check the performance of the sensors with different evaluation metrics (various evaluation metrics to test LCS are discussed in Section 6). If the performance (in terms of accuracy or precision) is not satisfactory, then repeat step 1, i.e., selection of sensors; otherwise, go to step 3.
Step 3: Calibrate the sensors in the field and evaluate their performance (Section 5.1.2). Once the field test is completed, they are ready to deploy in real-time in the field.
  Note: However, some studies have taken direct field evaluation steps without laboratory evaluation [48,49].
Step 4: Do frequent post-deployment analysis to check for data quality in real-time, which will help to identify the re-calibration requirement. In general, re-calibration is recommended to do at least once a month (Section 5.2).

## 3. Selection of Sensors

There is no particular definition for LCS. In general, devices which cost less than the reference grade instruments are considered LCS, but at-least a five fold cost decrements is expected. In the present commercial market, sensor devices that measure single parameters cost in between 10$–100$, and the multiple parameter monitoring devices at 100$–1000$. Sensors used for air pollution monitoring are divided into two categories:

1. Particulate matter sensors (PMS);
2. GAS sensors (GS).

### 3.1. PMS

PMS are used to monitor particles or aerosols, which are classified based on their size (diameter) as follows, PM_1_ (diameter ≤1 μm), PM_2.5_ (diameter ≤2.5 μm) and PM_10_ (diameter ≤10 μm). There are three methods to monitor PM, illustrated in the following points:

- Federal reference method (FRM): Gravimetric method is a FRM and it is a direct method to measure PM;
- Federal equivalent methods (FEM): Tapered element oscillating microbalance (TEOM), Beta Attenuation Monitor (BAM) are two different FEM methods to measure PM;
- Low-cost sensors: Most of the commercial particulate sensors are based on the light scattering principle, and few of them are work on other principles like digital holography and microscopy.

The first two methods are the reference methods discussed in detail in Table 2 and the third method is covered in the following subsections.

#### 3.1.1. Light Scattering Method

The majority of the low-cost PMS work on the light scattering principle, where the intensity of scattered light indicates concentration of the PM. In this technique, a targeted air sample is captured into the sensor’s hallow space. Light generated from the laser source interacted with the particles and scattered correspondingly to the size and count of the particles [52,53]. The photo detector at the receiver end converts the scattered light into an electrical signal. An algorithm is deployed to calculate the particle count by using the signal obtained from the photo detector. The maximum detectable particle size by this principle is ≥0.3 µm [54].

Advantages:

- Less cost and portable;
- Easy to operate and able to integrate with IOT network;
- High data resolution.

Limitations:

- PMS based on the light scattering principle can be used for particles of a size greater than 0.3 μm because particles of a size less than 0.3 μm may not scatter sufficient light to get particle count [54];
- Drift in the response due to the degradation of the laser source;
- Temperate and humidity can effect the sensor performance.

#### 3.1.2. Digital Holography Method

Major components in digital holography are optical signal generator, air sampling channel, image capturing system. Light generated at source passed through the continuous air sampling channel where light interacted with the particles. The imaging system at the receiver-end captures the interacted light and produces corresponding particles’ images in the sampled air [55]. Image reconstruction algorithms are used to count the number of particles.

Advantages:

- Able to detect particles of size in the lower μm-range, which is not possible in the light scattering method;
- It is an image based system hence there is no need to consider the flow rate monitoring;
- Possibility to find the chemical composition of particles by using the size and colour properties in the images.

Limitations:

- The sampling rate is less than that of sensors based on light scattering method.

#### 3.1.3. Microscopy Method

Major components in the microscopy sensors are CMOS (Complementary metal oxide semiconductor) imager, electrostatic particle collector, laser diode, Imaging substrate. At first, the electrostatic particle collector collects the PM on the imaging substrate and a glass slide can be used as the imaging substrate. Laser diode illuminates the substrate and the illuminated substrate is captured by the CMOS imagers. Image processing based PM sensing algorithm is used to convert the images into particle count and mass concentration [56,57].

Advantages:

- High volume sampling;
- Can detect sub-micron sized particles;
- Possible to detect Chemical characteristics of PM.

Limitations:

- Expensive compared to light scattering sensors.

### 3.2. Gas Sensors (GS)

GS sensors are used to measure gasses concentrations like O_3_, NO_2_, SO_2_, CO, CO_2_ etc. Most of the GS work on Metal oxide semiconductor (MOS) and Electrochemical (EC), two popular technologies in the commercial market. Non-dispersive infrared (NDIR) and photo-ionisation detectors (PID) are other rarely used technologies for GS making. Apart from these various advanced sensing materials such as graphene and derivatives of graphene, gallium nitride and carbon nanomaterials are explored to address reliability, response time and operating temperature limitations that existed in the presently available EC sensors [58]. However, there is a lack of studies on the evaluation of these advanced sensors for AQM.

#### 3.2.1. MOS Sensors

Metal oxide sensors capture the concentration of the pollutants on its metal oxide surface. Preheating of metal oxide surface is needed to capture the changes in the concentration of gaseous pollutants. The surface resistance varies corresponding to the concentration of pollutant in the samples, and that creates a proportional current flow in the circuit [59,60].

Advantages:

- They can work at higher temperatures and have a higher operational lifetime compared to EC sensors;
- High sensitivity;
- Less cost, portable and IoT integrable;
- High resolution data.

Limitations

- Heating of metal oxide surface is the limitation in MOS sensors. Pre-heating requires high operational power that makes MOS expensive in terms of power consumption;
- Higher humidity levels can reduce the metal oxide surface’s sensitivity, making the MOS less accurate when compared with ECS at higher humidity levels;
- Drift in the sensor performance due to the sensitivity loss of metal oxide surface overtime.

#### 3.2.2. EC Sensors

Electrochemical sensors (ECS) consist of of working electrodes and reference electrodes. When the gas sample diffuses into the sensors, it either oxides or reduces the working electrode and creates a potential difference between them that makes the flow of current proportional to the concentration. In addition to the primary electrodes, some sensors have one or more auxiliary electrodes to improve the sensitivity and stability of the sensors [61,62].

Advantages:

- ECS consume less power than MOS;
- High sensitivity;
- Less impacted by higher humidity values than the MOS sensors;
- Compact, portable and IoT integrable.

Limitations:

- Less operational time than ECS due to the degradation of electrolyte performance over time;
- ECS have a higher response time when compared to metal oxide sensors. In order to obtain the corresponding output to the applied input ECS undergo chemical reactions that cause the delay in response time.

#### 3.2.3. NDIR Sensors

NDIR sensors consist of an infrared red (IR) light lamp, a sampling tube, an IR detector and an optical filter. One end of the sampling tube is fitted with the IR lamp and the other end with the optical filter and the IR detector. The IR lamp directs the light towards the optical filter and detector through the sampling tube. When the air sample is pumped into the sampling tube, the IR light emitted from the IR lamp interacts with the gas and a portion of it is absorbed by the gas, and the remaining part hits the IR detector through the optical filter. The difference between the amount of light radiated by the IR lamp and the amount of IR light received by the detector is measured. Since the difference is the result of the light being absorbed by the gas molecules in the air inside the tube, it is directly proportional to the gas concentrations in the air sample. In general, the IR absorption is the best property of the CO_2_ [63], hence NDIR priciple is famous for CO_2_ sensor making.

Advantages:

- Compact and requires less power;
- High operational life time.

Limitations:

- Higher cost compared to ECS and MOS;
- Presence of moisture content in the air sample can cause the spectral interference that leads to inaccurate measurement.

#### 3.2.4. PID Sensors

PID sensors are familiar for volatile organic compounds (VOC) [39]. PID sensors consist of ultraviolet (UV) light source and associated electric circuits to capture the charge. The UV light ionizes the gas sample and creates charged gas molecules. The charged gas molecule constitutes a flow of current. The amount of current flow is directly proportional to the gas concentration [64].

Advantages:

- Low power requirements;
- High sensitivity and short response time.

Limitations:

- Very high cost;
- Difficult to design for a particular pollutant since UV light can ionize all the gases whose ionization potential is less than the energy of the UV light.

From the above discussion we conclude that each type has some advantages and certain limitations. In addition, before choosing any sensor, we need to consider various characteristics affecting sensor performance: rise time or response time, limit of detection (LOD), repeatability (ability to give same output under identical conditions), reproducibility (ability to produce same output under non identical conditions), environmental effects, sensitivity to other pollutants (cross sensitivity) are important characteristics of any sensor [33], explained in Table 3. Further temperature and humidity can also influence the LCS performance.

Hence, every aspect discussed above needs to be considered to adopt an LCS for AQM. Now the question arises on how to select a sensor out of the vast and complex information to set up an AQM with LCS? In fact, there is no standard procedure for this in the literature. By consolidating the analysis of various articles on LCS, we propose a procedure that can address the above question, illustrated in Supplementary Algorithm S1. This can reduce the cumbersome process of end-users in selecting LCS. Supplementary Figure S1 explains how our procedure can reduce the efforts in the selection of sensors.

## 4. Hardware Setup/Sensor Node Design

**Figure 4:**
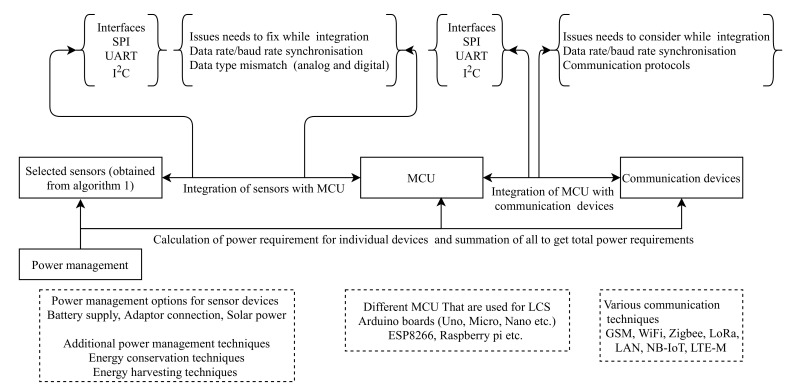
Hardware setup preparation with LCS for AQM.

Hardware setup/sensor node design can be implemented in three stages as follows, and illustrated in Figure 4.

- Integration of sensors with MCU;
- Integration of MCU with communication devices;
- Power management

In addition, it is needed to adopt various quality control mechanisms in the hardware implementation, which is covered in the next section.

### 4.1. Integration of Sensors with MCU

Selected sensors need to be interfaced with a microcontroller (MCU) for further processing before data transmission. In general, sensors interface with MCU by using Serial peripheral interface (SPI), Inter-Integrated Circuit (*I*^2^*C*), Universal Asynchronous Receiver Transmitter (UART) [66,67]. For example, the SDS011(PM sensor from Novafitness), PMS5003M (PM sensor from Palntower) use the UART interface [68,69]. Whereas, OPC-R1, OPC-N2 (PM sensors from Alphasense) use SPI interface [70,71]. UR100CD (temperature, humidity sensor from Technosens) and BMP180 (pressure, altitude sensor from Bosch) interfaced with *I*^2^*C*. Some of the sensors have analog outputs. In order to make sensor output compatible with MCU, an analog to digital conversion is needed. For instance, Alhasa et al. and Zimmerman et al., converted the analog output of AlphaSense sensors before integrating with an MCU [20,72].

Another issue to be considered while integrating sensors with MCU is baud rate synchronization between sensor and MCU. Verifying baud rates and programming MCU accordingly eliminates the data loss. Most of the LCS have a baud rate of 9600 [68,69,73]. Fixed sampling rate is a better option in order to use heterogeneous baud rated sensors. With fixed sampling rate, we can enable periodic wake-up of the sensor node, useful for low power operation [21]. Furthermore, with the full swing of IoT and industry 4.0 various development boards are readily available for sensor integration, making the task of integration and compatibility easy [74]. Arduino boards [24,75,76,77,78,79,80,81], ESP8266 [49,82,83], Raspberry Pi [56,63,65,72,82,84,85,86] are some of the development boards extensively used to develop AQM sensor devices.

### 4.2. Power Management

Power management in the node is another crucial aspect. For heterogeneous operating voltages of sensors and other associated components, we need voltage level shifters, and voltage regulators [87]. It is helpful to calculate the power required to the entire node to make better power management. Adapter connection, battery supply, solar power are different options to power the sensor node. Battery operated nodes are flexible with location and more suitable for mobile applications. However, these nodes have limited operational power due to the usage of batteries that are of limited capacity. To improve battery lifetime, we can incorporate various energy harvesting options such as thermal, vibrational, solar and wireless energy harvesting techniques in the sensor node [88]. Different energy conservation techniques like MAC (Medium Access Control) layer scheduling of transceivers, reducing the overhead of the protocols and incorporating power management schemes further enhance efficiency in power utilization [89,90].

### 4.3. Integration of Sensors with Communication Devices

Connecting the sensor nodes to the communication backhaul is unavoidable to access data remotely. GSM, WiFi, and dedicated LAN (local area network) connection are helpful for data logging into the remote server. Among GSM, WiFi and LAN, GSM is very popular in the sensor node communication since the other two have very limited access in remote areas. For instance, Brzozowski et al. [91] deployed the PM monitoring sensor nodes at the road intersection and the data is communicated to the server with 3G, GSM. In another study, Hasenfratz et al. [25] used both GSM and WiFi to send the LCS data to a remote server to create high density urban air pollution maps. Zigbee (IEE 802.15.4), Bluetooth are suitable for short-distance communication that can be considered for inter-node communication and data transfer to a gateway in a sensor network. Rasyid et al., sent sensor data to a nearby gateway with the help of Zigbee as a part of their gas sensor network communication [80]. Low power Radio (LoRa) is a recent advancement of radio communication especially designed for IoT devices [92]. It has the features of low power, long-range, and high security required for the wireless sensors network. We believe that LoRa can enhance the power management and remote communication of LCS devices [21]. Furthermore, newly incorporated NB-IoT and LTE-M low power wide area communication technologies in 5G are helpful for battery operated low power devices such as LCS based AQM devices. Hence, users can adopt the communication technologies mentioned above in air quality sensor devices based on their applications. Micro-SD cards are helpful for in-device data logging purposes that can tackle sensor devices’ temporary connection failure to the backhaul communication network. Table 4 illustrates various communication techniques that are useful in sensor node design.

Further, a robust hardware prototype is recommended for field deployments to avoid internal components’ disintegration due to extreme environmental conditions like high wind and thunderstorms. In general, sensors have an operational lifetime. Operational lifetime can be defined as the time duration that a sensor can work within the prescribed levels of accuracy. Manufacturer provides the operational lifetime on the sensor data sheet. However there is a lack of credibility on the manufacturer information. The following procedure can help to find out the operational lifetime of a sensor.

- Sensors’ operational lifetime depends on the lifetime of internal components of the sensors. The lifetime that is least among the internal components of a sensor is the operational lifetime of that sensor. For example, an optical sensor’s lifetime depends on a light emitting source (laser source) and photo detector. Therefore the light emitting or photo diode which has the least operational lifetime is considered the operational lifetime of the optical sensor.

Once the life period is completed, the sensor has to be replaced with a new one. Now the question arises; if the sensor can work until the completion of operational lifetime or we need to replace prior to that, how can we check the declining nature of sensor performance? The following discussion can be useful to identify sensor replacement prior to the end of operational lifetime.

- LCS performance drifts overtime. Main reason for the sensor response drift is the degradation of sensing material or sensing mechanism. This drift can be clearly visible in the time series or trend lines. Therefore identification of unusual drifts in the sensor output signals the replacement of the sensor prior to the end of operational lifetime;
- Sensor output trend reversal when compared with reference instruments values and continuous outliers also helps to recognize the requirement of sensor replacement.

## 5. Calibration and Evaluation

Calibration and Evaluation are the most important steps in order to establish a sustainable low cost AQM setup. To avoid an abrupt jump into the calibration and evaluation, we started with basic terminology that can make readers more understandable.

Sensors capture a physical phenomenon and produce corresponding electrical signal variations (voltage or current) at the output. Manufacturers map such variations to the corresponding concentration of the pollutants and provide a nominal data-sheet. In other words, we call these mappings the sensor’s raw output. Several studies reported serious inaccuracies while using these raw outputs in real-time due to various error sources such as low selectivity, limit of detection, non-linearity, bias and offset, environmental effects, signal drift etc. We recommend the study by Maag et al., to understand various error sources affecting the performance of LCS for air pollution measurement [37].

In order to tackle the error sources mentioned above, researchers have suggested a calibration process that transforms the raw output to the corresponding reference-grade instrument values. Numerous studies have reported significant improvement in the accuracy with the calibration process [23,75,84,85,93,94,95,96,97,98]; single variable regression, multiple variable linear regression, polynomial regression, random forests, k-nearest neighbours, artificial neural networks are some of the models already used for LCS calibration [20,99,100,101]. Calibration needs to be done both before deployment (pre-deployment) and after deployment (post-deployment)

### 5.1. Pre-Deployment Calibration and Evaluation

Pre-deployment calibration has to be done at two places:

1. At the laboratory with controlled environmental conditions by using standard gaseous mixtures;
2. In the field with uncontrolled real-time environmental effects.

#### 5.1.1. Laboratory Calibration and Evaluation

Laboratory calibration involves exposing LCS to different concentrations of targeted pollutants under a controlled environment (temperature and humidity) inside a chamber. For example, Cheng et al., used a 10 m^3^ cubic glass chamber with an air conditioner inside to control temperature and humidity and air purifiers to vary PM concentrations [102]. However, they did not mention the procedure to get different PM concentrations with their setup. At the same time, it is not easy to generate stable PM concentrations in cubic chambers [75,76,103,104]. In order to obtain a uniform PM concentration, Sayahi et al., designed a cylindrical chamber with a controlled environment and tested eight PMS3003 low-cost PM Sensors [104]. Masson et al., enclosed an array of CO sensors (MiCS-5525 from Sensortech) in an aluminium box and fixed them to a mixing manifold inlet [59]. They used a duty-cycle controlled heat lamp for temperature adjustment and flow controllers for gas concentration adjustments.

In contrast to using the same chamber for all parameters, Wei et al., used different setups for temperature and humidity to calibrate electrochemical gas sensors [85]. To the best of our knowledge, only one study developed a laboratory calibration setup for both particulates and gasses [103]. At the same time, we identified a lack of mass laboratory calibration procedures to calibrate a high volume of sensors.

**Figure 5:**
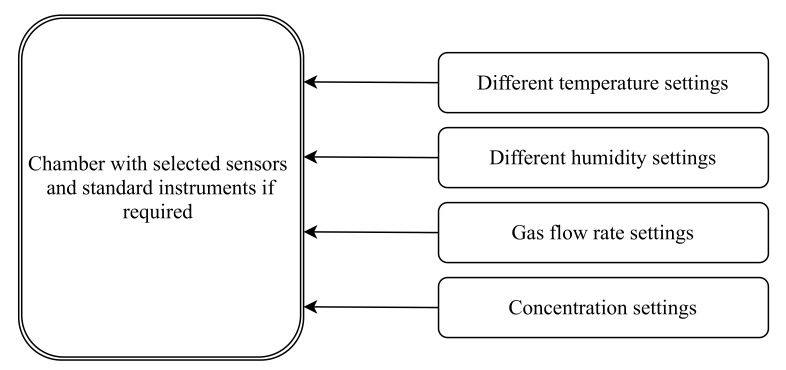
Typical laboratory calibration setup for LCS.

Once the sensors are placed inside the calibration chamber, apply the targeted concentrations with controlled environmental conditions and record the output values of the sensors. The recorded values are used to fit a model with the original concentrations termed as a calibration model. In general, laboratory calibration deals with the sensing principles of the sensors [37]. At this stage, different characteristics of sensors such as linearity, accuracy, cross-sensitivity and effect of temperature and humidity are analyzed and corrected [59,64,76,85,105]. Furthermore, we can check for the repeatability and reproducibility of the sensors [65]. Figure 5 shows a typical block diagram of a laboratory calibration setup. Table 5 and Table 6 cover various laboratory calibration procedures followed to evaluate sensors in the laboratory.

Laboratory calibration alone is not enough to deploy sensors in real-time since it does not reflect all the characteristics of a specific location that they will be deployed [65]. For example, Zamora et al., reported severe inaccuracies in the field measurement even though they did the laboratory calibration [106]. Hence, in-field calibration is a necessary step following laboratory calibration. Several recent studies explored direct infield calibration without laboratory evaluation and reported good agreement between the sensor and standard methods [48,49,107].

#### 5.1.2. In-Field Calibration and Validation

In-field calibration needs the collocation of the sensors with standard devices. We can achieve this in two ways: (1) Keeping sensors next to the AQM stations handled by regulatory bodies [24,121,122,123]; (2) Using reference-grade instruments equipped on a vehicle [63,84,124]. The former approach is only possible if reference stations are available near the location of interest. If no reference station is available, the latter approach is useful. Transfer calibration (transfer of calibration parameters of one sensor to another sensor) is another possible approach for LCS in-field calibration when reference stations are not available nearby. In general, transfer calibration is used in the sensor manufacturing industries for mass calibration with the help of one master sensor. Recently Cheng et al., explored the transfer calibration technique to calibrate air pollution network established in Beijing and surrounding cities [125]. It is a popular technique for electronic nose calibration that is used to detect odours and flavours in the chemical industry [126,127].

Once the sensor devices and the reference instruments are collocated at a place of interest, the pair wise data obtained from them is used to fit a model [128,129]. for example $S_{i}^{\left(NX1\right)}=\left\{s_{1},s_{2},...s_{n}\right\}$ is a set of outputs from sensor *i* with *N* samples and $R_{i}^{\left(NX1\right)}=\left\{r_{1},r_{2},...r_{n}\right\}$ is the corresponding set of the reference instrument values then model *C* which transfers *S* values to *R* values is the calibration model, that is,

(1) R=C(S).

Sensor nodes have to be collocated with AQM stations such that both are exposed to the same environment. Inlets of the LCS devices and reference-grade instruments should be very near and at the same height. At the same time, maintain inlets at a certain height from the ground level [27]. In order to maintain the collocation requirement of both inlets at the same height, sensors are placed on the AQM station’s rooftop. The distance between the inlets is from 1 m to 4 m. However, the height difference of the inlets is not available. According to our knowledge, it is not more than 4 m [85,94,130,131,132]. Zheng et al., strapped the sensor boxes to a tripod of the reference-grade instrument to achieve a better collocation distance [133]. In contrast, Trilles et al., used a mobile vehicle and fixed the inlets on the vehicle’s top. Crilley et al., reported performance comparison difficulty due to bending in the inlet tube of TSI 3330 (a reference grade instrument) [86]. Hence, we can conclude that the sensor devices and the reference-grade instruments must be exposed to the same concentrations to fit a better calibration model.

Once the pairwise collocated data are available, we can fit a calibration model mentioned in Equation (1). Data-driven approaches such as machine learning and artificial intelligence outperform in-field calibration because they can identify the complex air pollution data’s underlying patterns. For example, Johnson et al., tested single variable linear regression, multiple variable linear regression and gradient boosting regression models for PPD42 PM sensors and found that the latter one out performs the former [84]. Cross et al., used higher dimensional model representation (HDMR) for NO and CO sensors calibration in their university campus [23]. Table 7 illustrates the various calibration models used in previous studies. In the same table, we highlighted the outperforming models in their respective studies.

Location and background of the site are also crucial considerations while doing in-field calibration. We found that the same sensors are calibrated with different calibration models when deployed in other locations. For instance, Stavroulas et al. [99], and Zheng et al. [133] used polynomial regression to calibrate PMS5003 (PMS) at Athens (Greece) and Durham (UK). In contrast, Minxing et al. [131] used a feed-forward neural network in Calgary (Canada) for the same sensor. In Table 7, we can identify more such examples. Furthermore, Bigi et al., showed that support vector regression (SVR) and Random Forest (RF) techniques are better suited than the linear models in an urban background with high vehicular movement. Hence, users can also pay attention to the location and the background of the site for a better in-field calibration model.

Discussion and Results: Suppose a manufacturer provides enough laboratory evaluation and analysis to the users and the manufacturer is reliable, only then we can go directly to in-field calibration. Otherwise, we need to perform laboratory evaluation to understand sensor characteristics. In addition to Environmental effects and cross sensitivities, we need to consider the site’s background for in-field calibration. So far there is no harmonization in the calibration approaches for LCS. Hence, choosing a better performing model is based on sensors selected and other factors discussed above.

### 5.2. Post-Deployment Calibration and Evaluation

One of the main drawbacks of the LCS is the lack of reliability of the data [27]. Though sensors undergo rigorous evaluations in the lab and field, data reliability is questionable for long duration deployments due to sensor signal drift. Frequent recalibration (post-deployment calibration) can address this issue. However, it is is not possible to maintain reference stations everywhere. Hence, various calibration strategies; Blind calibration, Collaborative calibration, Transfer calibration are explored for post-deployment calibration and illustrated in Table 8. Cheng et al., proposed transfer calibration for post-deployment calibration by using calibration parameters of one sensor at some location to another sensor at another site [125]. However, in order to perform transfer calibration, both the places must have less divergence in the pollutants distributions. The same study reported a maximum duration of one month is feasible for transfer calibration. We recommend more precise and identical sensors for this kind of calibration. A detailed study by considering different places and different durations is one of the requirements in this aspect.

Multi-hop calibration is another approach, where already calibrated sensors against reference station fixed on a vehicle used to calibrate other sensor nodes in the network. Error accumulation over the nodes in long networks is the problem associated with this. Different statistical method were developed to address this error accumulation problem [134,135]. Furthermore Barcelo et al., suggested on-line or remote calibration [24]. Remote calibration is achieved in two ways, as follows:

1. By replacing the calibration parameters in the sensor nodes. In order to do this, we need to incorporate the re-calibration mechanism within the sensor node, which requires high computational power. At the same time, it is not feasible to handle extensive data at the sensors node;
2. Doing calibration in the cloud by taking raw sensor data as the inputs. With this technique we can overcome the limitations in the former method.

In addition to the re-calibration, quality assurance (QA) and quality checks (QC) are also needed for reliable data in air quality measurements [136].

QA is needed for reducing the occurrence of errors while measuring and QC for identifying the erroneous data after measuring. QA deals with various hardware and software issues while measuring with LCS. Loss of power and internet, disintegration of hardware components in the nodes are the primary hardware issues [87,137]. For instance, Zheng et al., lost the meteorological data occasionally due to power failure [133]. Maag et al., were not able to calibrate all the sensor nodes in the network due to irregular schedules in their multi-hop calibration [134]. Similar problems were reported by Kizel et al., during the relocation of senors nodes [135]. Bun et al., experienced the loss of data for specific periods due to intermittent internet connection [138]. To deal with the intermittent connection, Becnel et al. [21] use local storage (SD card). When the connection is resumed, locally stored data are automatically compared with the database and are replaced the missing data.

Various data recovery approaches such as spatial correlation, Markov random field model and compressed sensing are available in the wireless sensors networks (WSN) to handle the missing data [139,140,141]. However, we cannot say that these replaced values are exactly equal to original lost values. So this approach may be the last priority in data handling techniques for LCS. As per our knowledge, no studies on LCS for air pollution measurement has been reported using such methods.

Frequency mismatch while measuring and posting data, improper conversion of analog signal to digital data, capturing heterogeneous data generated by different sensors are some of the software related issues.

Sensor data have to undergo various quality checks before disseminating to the public. Identification of sudden peak or valley, missing data, and persistence of calibration parameters are various quality checks. Removing or replacing the outliers is a primary quality control. We can achieve this by comparing sensor data with reference stations at regular intervals. For instance, Campbell et al., suggested flagging of suspected data for the quality checks [136]. Bulot et al., used six levels of quality checks by keeping certain thresholds at each level [132]. We can do QA & QC at the sensor nodes or in the cloud by deploying an automated process or with regular manual intervention. Manual verification is a cumbersome process for big data, and therefore automatic flagging is preferred.

## 6. Evaluation Metrics

Numerous commercial LCS are available in the market for AQM. However, sparse information provided by manufacturers on data-sheet makes users lose confidence in the usage of LCS. At the same time, lack of complete guidelines from the regulatory bodies on LCS usage makes the situation further difficult. Hence, the selected sensors need to undergo evaluation before using in field [28]. Various evaluation metrics are used in the literature for LCS data validation. We conclude from the existing studies that evaluation should be made in three scenarios to ensure the sensors data accuracy and reliability. (1) Evaluation of the sensors against reference station for accuracy (sensor vs. reference) [20,22,23,24,25,27,79,84,93,94,100,120,123,125,135,153]. (2) Evaluation of sensor against the same type of sensor for precision (sensor vs. sensor) [49,93,95,100,133,154]. (3) Comparison of different models performance to finalize better one (model vs. model) [20,21,122].

### 6.1. Sensor vs. Reference

In this scenario, sensors are evaluated against the reference instruments values. correlation coefficient (*r*) and spearman’s correlation coefficient (*ρ*) are the metrics to check degree of agreement between the sensor values and reference values [79,93,120]. The former metric indicates only a linear relationship, whereas the latter indicates a monotonic or affine relationship. In fact, LCS does not have a perfect linear relationship with reference values in maximum instances. So, *ρ* is the most suitable parameter. coefficient of determination (*R*^2^), mean absolute error (MAE), root mean squared error (RMSE), normalized RMSE (nRMSE), centered RMSE (CRMSE), mean bias error (MBE), coefficient of efficiency (COE) are used to analyze error in the sensor data [27,84,94,123,125]. Measurement uncertainty (U) accounts for all types of errors while measuring with LCS [28,117,121,155]. It is an approved metric by European air quality directives [156]. However, it is not included in much of the existing literature on LCS due to difficulty in calculation [34]. Quantile-quantile (QQ) plot shows better visualisation of deviation of sensor values from the reference measurements [22]. Match score analysis between reference values and sensor values are useful for air quality indication at a coarse level and can be used for awareness and education of people towards air pollution [117].

### 6.2. Sensor vs. Sensor

This evaluation aims to verify the consistency in the data produced by sensors, also referred to as precision. Precision is expressed in terms of repeatability and reproducibility [33]. Coefficient of variation used as a metric to test repeatability and reproducibility of LCS [65,132,133]. In addition to this, correlation metrics, covariance metrics also included in the studies to express consistency among sensors [95,100,154]. Furthermore, normalized root mean square error (nRMSE) is also explored to validate reproducibility of sensors [49,105].

### 6.3. Model vs. Model

As we discussed earlier, a better calibration fit improves the accuracy of LCS data. However, there is a gap in the harmonization of calibration approaches. Therefore, fitting a better model is based on verifying different models for the given set of conditions in real-time. To do that, various metrics are used to opt for a better model; slope and intercept values are used to check the linearity of fits [21,22,79]. *R*^2^, MAE, MBE, RMSE nRMSE can be used to compare different models [23,84,153]. For example, *RMSE*_1_,...*RMSE_i_* and *MAE*_1_,...*MAE_i_* are the root mean squared values and mean absolute values of the models 1 to i then the model performing best in both metrics can be opted as outperforming model. However, Comparing more than one metric at a time is a cumbersome process. Target diagrams, Bland-Altman plots can be used for comparing more than one metric to choose a better model [20,21,122].

Therefore, testing sensors with at least one reliable metric in each scenario is necessary to get accurate data from the LCS. Comparing different models is the user requirement based on the number of models they opted for testing. Supplementary Tables S1 and S2 illustrate various metrics for the evaluation of LCS in different scenarios.

## 7. End User Applications

**Figure 6:**
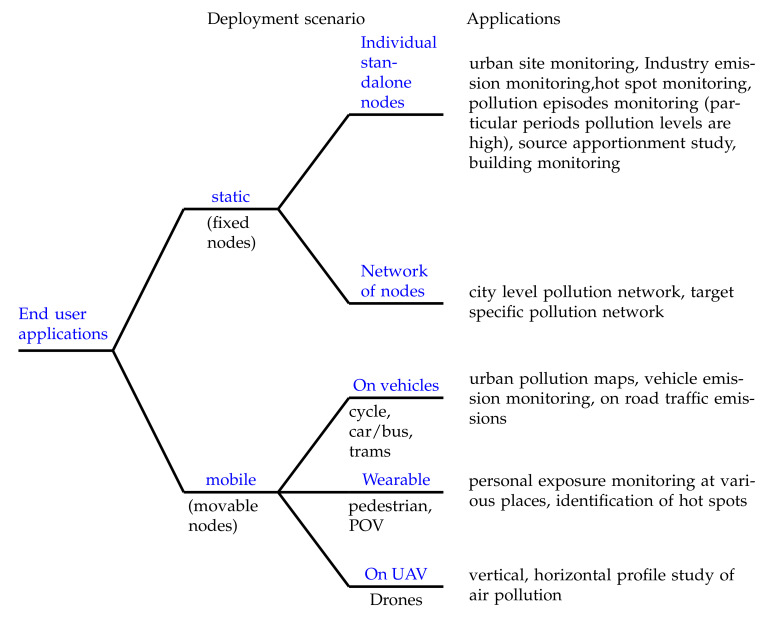
End-user applications of LCS. Various studies that are used LCS devices; for individual standalone node applications are [91,117,120,132,154,157,158,159,160,161,162,163]; network of nodes applications are [21,62,79,80,119,143,164,165,166,167,168]; on vehicles applications are [25,81,120,168,169,170,171,172]; wearable applications are [15,16,62,81,173,174,175,176], and on UAV applications are [36,177,178,179,180,181,182]. Here, POV stands for person on vehicle (cycle, motor bike etc.)

This section discusses various applications of the LCS in AQM. We divided LCS applications into two categories; (1) Static deployments (2) Mobile deployments, shown in Figure 6 based on deployment in the field. Further, sensor devices can be deployed as a standalone individual node or as network of nodes.

### 7.1. Static Deployment

In static deployments LCS are placed in a fixed place throughout the monitoring period. Industry emission monitoring, urban site monitoring, building monitoring, source apportionment study are examples of stationary deployments [21,62,79,80,91,117,119,120,132,143,154,157,158,159,160,161,162,163,164,165,166,167,168]. Castell et al., monitored air quality at kinder-gardens in Oslo city, Norway [157]. A similar study was done by Bulot et al., for AQM at schools in Southampton city, UK [132]. Tsujita et al., created a sensor network at Tokyo Institute of Technology, Japan for gaseous pollutants monitoring [143]. In a similar study, Mead et al., created a sensor network in their university for air quality monitoring [62].

### 7.2. Mobile Deployments

In contrast to static deployments, LCS are mounted on vehicles in case of mobile deployments [183]. Vehicular emission monitoring, air pollution mapping with the vehicular movement, vertical profile study of air pollution using drones are examples of mobile deployments [15,16,25,36,62,81,120,168,169,170,171,172,173,174,175,176,177,178,180,181,182]. Very frequent calibration is needed in this case due to sensor exposures to various environments, introducing different bias values in the calibration model. Wearable is a special case in mobile deployments where the LCS are worn by persons for personal exposure monitoring [15,16]. Miniaturization and less operational power are important requirements in this case [175]. Non linear calibration models are more feasible for wearable LCS devices [174].

Unmanned aerial vehicle (drone) equipped with LCS are used to study the higher dimensional air pollution profile (horizontal and vertical directions) [180,181]. Turbulence effect on sensor inlet airflow, electronic interference from drone operation, changing pressure values with altitude, vibrations, tilting of sensors during the flight are possible additional errors in this case [36]. As mentioned earlier, collocated data of LCS and reference devices are needed for calibration, which is impossible here since carrying heavier reference equipment on drones is not feasible. Limited studies have explored the usage of LCS on drones and reported inaccuracies [178,182]. Since there are additional error sources mentioned above, we believe the calibration methods explored so far may not be applicable in the case of UAVs. We can identify a need for accuracy improvement procedures to use LCS on UAV. The inclusion of additional error sources while calibration and advanced sensors resilient to vibrations and electronic interference can be a better choice for UAV applications. Researchers can work on this open problem of accuracy improvement of LCS on drones/UAVs. Figure 6 illustrates the stratification of end-user applications using LCS.

## 8. Conclusions and Future Scope

LCS made a significant improvement in air quality monitoring at a fine scale. LCS data can be used as a supplement to the conventional methods of AQM. However, policymakers have not adopted this method due to poor data reliability. One of the reasons for this is the lack of long-duration evaluation and exact quantification of errors. Even though there are studies on LCS evaluation for AQM only a few reported adequate accuracy levels. Lack of standard protocols for calibration and performance evaluation also hinder the adoption of LCS. AQM framework and critical analysis at each step in this study make LCS users’ task easy.

In the case of sensor selection, the procedure we propose can help end-user to make a proper selection effortlessly. MOS have higher power consumption than EC. However, they can work at higher temperatures. At the same time, MOS have a higher response time compared to EC. Existing PMS on optical principle are not suitable for particles of diameter less than 0.3 μm. Advanced sensors mentioned in the selection of sensors can be explored for this application. Present LCS have an operational lifetime of one to one and a half years. Hence there is a need for the development of sensors with a high operational lifetime.

Sensor performance is location and application dependent. Finding a model that suits every scenario is impossible. Hence, users need to identify the possible error sources in their application and counter them with calibration and frequent re-calibration. Performance evaluation of sensors and calibration models are unavoidable to achieve higher accuracy. Adopting QA and QC can further improve data reliability that builds users’ trust in air quality measurement with LCS. Fitting a universal calibration model is not possible due to heterogeneity in the environmental conditions and sensors. There is a lack of mass calibration procedures to calibrate and evaluate higher volumes of sensors in the field. Only a few studies have explored calibration methods for post-deployment calibration when reference stations are not available. Hence, post-deployment calibration methods need to be developed to calibrate an entire network without dislocating the sensor devices. A combination of data-driven approaches and statistical models may be suitable in this regard. Calibration at regular intervals addresses the sensor’s drift over time. However, regular interval calibration at remote areas is cumbersome. Remote calibration methods may be another potential area to work to reduce the tedious procedure of re-calibration.

Since there is no harmonization of performance criteria, LCS needs to be evaluated with at least one reliable metric in each scenario mentioned in the evaluation metrics before use in real-time. Prior QA/QC is needed to release LCS data in real time.

## Figures and Tables

**Table 1:** Various surveys/reviews on LCS for AQM. Here, LCS: low-cost sensors, AQM: air quality monitoring, IAQ: indoor air quality, VOC: volatile organic compounds.

Authors	Survey/Review
Rai et al. [33]	Comprehensively reviewed various critical aspects in the LCS’s performance assessment.
Karagulian et al. [34]	Presented a detailed study on the performance of various commercially available LCS.
Kumar et al. [35]	Reviewed the benefits and challenges of using LCS in indoor AQM.
Hedworth et al. [36]	Studied the effectiveness of LCS on drones for AQM.
Maag et al. [37]	Summarized various error sources that influence the performance of LCS and calibration approaches to counteract the errors.
Kumar et al. [38]	Consolidated the challenges while using LCS in urban environments.
Chojer et al. [19]	Summarized the developments in IAQ monitoring devices with LCS.
Zhang et al. [39]	Reviewed the guidelines of various environmental regulatory agencies for IAQ and characteristics of LCS that are useful in IAQ monitoring.
Borrego et al. [40,41]	Validated various LCS against reference stations.
Aleixandre et al. [42]	Reviewed the performance of commercially available gas sensors.
Morawska et al. [18]	Reviewed the suitability of LCS for various applications and improvements needed further to adopt them in full potential.
Concas et al. [43]	Reported and analyzed various machine learning algorithms used to calibrate LCS.
Alfano et al. [44]	Comprehensively listed and analyzed the different PMS and their performance in AQM.
McKercher et al. [45]	Reviewed the performance of different low-cost air quality monitors that usages LCS.
Thompson [46]	Comprehensively reported and analysed various sensing technologies that are useful in manufacturing LCS.
Spinelle et al. [47]	Reviewed the performance of various Benzene and VOC sensors.

**Table 2:** FRM and FEM methods.

Sensing Principle	Advantages	Limitations
Gravimetric methodology: Sampled air passed on to the filers made of quartz or glass or Teflon The filter is weighed on an analytical weighing balance before and after sampling Weight difference divided by the volume of the gas pumped into the samplers for 24 h gives the PM value in $\mu g/m^{3}$	It is an absolute method and involves direct method of measurement Accurate method of measurement Less prone to electronic and mechanical noise interference.	Not possible to get continuous data in real-time. Only 24 h sampling period is available. More tedious due to the involvement of filter weighing
Tapered element oscillating microbalance (TEOM): Air sample passed on to a tapered element. The tapered element consists of a oscillating mechanism vibrates at its natural frequency Accumulation of particles on tapered element changes the natural frequency of oscillating mechanism which is converted as particle concentration.	Continuous measurement is possible Cost effective and labour effective in comparison with gravimetric methods	Higher temperature and humidity can damage the sensitivity of micro balance [50] Sensitive to electronic and mechanical noise interference Initial calibration is required
Beta Attenuation Monitor (BAM): Sampled air passed on a filter tape, which was associated with a beta ray emission facility (carbon-14 element) on top and beta ray detector at bottom. Beta rays are counted before and after the air sample. The number of beta rays attenuated tells the particle concentration.	Continuous monitoring is possible Easy to operate compared with other methods	Not a direct method of measurement and required initial calibration with standard dust Adsorbed water content in the sample can effect the measurement [51].

**Table 3:** Various characteristics of LCS.

Characteristics	Definition	Measurement Methods
Sensitivity (*S*)	Sensitivity refers to the slope of the input-output characteristics curve of a sensor under steady-state operation. It indicates how the output varies to the corresponding change in input. It is constant if the sensor has liner characteristics; otherwise, it will change as the input changes. In general, voltage or current is the output of LCS and pollutant concentration is the input. For example, OX-B431 Sensor (O_3_ sensor, Alphasense company) has a sensitivity of (−225 to −750) nA/PPM	$S=\frac{\Delta y}{\Delta x}$ y is output & x is input of a sensor
Range (*R*)	Range is defined as the maximum and minimum values of the input that a sensor can recognize. Sensor operation beyond the range can produce erroneous output	*R* = (*x_max_*, *x_min_*); *x_max_* is maximum value of input & *x_min_* is minimum value of input
Accuracy (*A*)	Accuracy indicates the closeness of the sensor reading with the corresponding reference instrument value.	$A=1-e=1-\frac{\left(s-r\right)}{r}$; *s* is sensor reading & *r* is reference instrument reading
Reproducibility	Reproducibility indicates consistency in the sensor output for the same input. Some studies have considered coefficient of variation (*CV*) as a metric to measure the reproducibility [65].	$CV=\frac{\sigma }{\mu }$; *σ* is standard deviation & *μ* is mean of sensor readings
Response time (*t*_90_)	Response time is defined as time taken for the sensor to reach 90% of it’s stable input value	*t*_90_ = *T* (0% of *x* to 90% of *x*); *x* is the input value to a sensor
Selectivity	Selectivity indicates how the sensor performs in the presence of other inter-fearing pollutants. For example, the NO_2_ gas sensor is often sensitive to O_3_, that means the presence of O_3_ affects the performance of NO_2_ sensor, and this is also called as NO_2_ sensor cross-sensitive to O_3_ [29].	The cross-sensitivity of a sensor can be calculated by exposing sensor to the other pollutants

**Table 4:** Various communication techniques that are useful in AQM sensor node design.

Standard	Date rate	Range	Operating Frequency	Advantages	Limitations
GSM	kbps to several hundred Mbps	10–15 km (2G), 1–2 km (4G)	169 MHz, 434 MHz, 470 MHz, 868 MHz and 915 MHz	High range	Low data rate
WiFi (802.11)	10 Mbps to 100 Mbps	100 m	2.4/5 GHz	High speed High data rate	Short range High interference
LoRa	10 kbps to 50 kbps	10 km to 20 km	169 MHz, 434 MHz, 470 MHz, 868 MHz and 915 MHz	Low power requirement Designed for IoT High range	Low transmission rate
Bluetooth (802.15.1)	125 kbps to 3 Mbps	10 m	2.4 GHz	Less power requirement Easy to connect	Very short range No security
Zigbee (802.15.4)	20 kbps to 250 kbps	10 m–100 m	2.4 GHz	Low power Less cost	Low transmission rate Low range Insecure

**Table 5:** Various laboratory calibration setups for particulate matter sensors based on the light scattering principle.

Sensors Tested (Manufacturer)	Calibration Setup Details	Authors
PMS5003 (Plantower), SDS011 (Novafitness), SPS30 (Sensirion), GP2Y1010AU0F (Sharp), PPD42NS (Shinyei), B5W-LD0101 (Omron)	Sensors were enclosed in a chamber that was connected with a stable PM generation facility. Particles were generated by dissolving a non-volatile solute with a volatile liquid in a vibrating orifice aerosol generator 3450 (VOAG, TSI Inc., USA). Particles were neutralized before injecting into the chamber by using a charge neutralizer. Dioctyl sebacate (DOS, density of 0.914 g/cm${}^{3}$) was used as the non-volatile solute and 2-propanol solvent (>99.999%, Sigma-Aldrich) as the volatile liquid. A programmed gradient pump (GP50) was used to generate different concentrations of non-volatile liquid, and uniform droplet sizes were obtained with the help of VOAG.	Kuula et al. [108]
GP2Y1010AU0F (Sharp), PPD42NS (Shinyei), DSM501A (Samyoung), CP-15-A4 (Oneair)	Sensors were mounted vertically inside a cylindrical chamber made with Plexiglas. Particles were generated using three different devices: stainless steel atomizer, up-drifting nebulizer, and dust generator (TOPAS SAG 410/U). The generated particles were injected into the chamber with the help of a 4${}^{\prime \prime }$ way PVC connector. Sodium Chloride, Methylene blue and Fluorescein sodium solutions were used to produce particles inside the atomizer. The up-drifting nebulizer was used to generate particles of different size distributions, and different standard deviations were achieved with the collision atomizer.	Liu et al. [77]
PMS5003 (Plantower)	Sensors were placed inside a test chamber (volume approximately 50 L), and the particles were injected into the chamber. A nebulizer was used as a particle generator, and that was connected to the chamber in series with a dryer, neutralizer (TSI 3077a) and a differential mobility analyzer (DMA). Neutralizer neutralizes the charged particles, and the DMA was used for the size selection of particles. Condensation Particle Counter (CPC, TSI 3786). A Wide Range Particle Spectrometer (WPS, MSP Corp.) and an Aerodynamic Particle Sizer (APS, TSI 3321) were used as reference instruments.	He et al. [78]
OPC-N2 (AlphaSense)	Sensors were enclosed inside a rectangular box. Particles of size greater than 2.5 μm are generated with the help of loud speaker and particles of size less than 2.5 μm are generated with smoke generator. Uniform temperature of approximately 20 ${}^{\circ }$C and relative humidity of 50% is maintained throughout the experiment. Grim is used as a standard instrument and placed near the sensors at bottom of the box.	Jagatha et al. [109]
PPD42NJ (Shinyei)	Sensors were placed inside a specially designed 10 m${}^{3}$ air chamber with full climate control. Air conditioner is used to control temperature and humidity. Due to large size of the chamber it is possible to calibrate 16 sensors at a time. The particle concentrations are varied manually. However, it is not clear how they generated particles	Cheng et al. [102]
PPD42NJ (Shinyei)	Two separate chambers, a mixing chamber and a sensor holding box were used to calibrate four PPD42NJ particulate sensors. At first, particulates with dry filtered air were injected into the mixing chamber with the help of a nebulizer and a steel tube. Mono-disperse polystyrene spheres and poly-disperse dust were used as the particulate sources. Fans were used inside the chamber to make particles suspend inside the chamber. Then, the sensor holding box was placed in series with the TSI APS (Aerodynamic Particle Sizer) inlet connected with the mixing chamber to suck the particles. The reading of the sensors and APS were tabulated until the concentration reached a specific limit to calibrate the sensors.	Austin et al. [75]
GP2Y1010AU0F (Sharp), ZH03A (Winsen), SDS011 (Novafitness)	Test sensors were placed inside a chamber made with an acrylic sheet, and each side was glued such that there were no leakages. A condensation particle counter, TSI 3025A and a Honeywell pre-calibrated particle sensor (HPMA115S0-XXX) were used as reference devices. The sensors and reference devices were placed adjacently inside the chamber. incense sticks were used as the particulate generators, and generated particulates were pumped into the chamber through silica gel, buffer, pressure regulator, and HEPA filter to provide dry, stable, and clean airflow.	Hapidin et al. [110]
Not available (Total 264 sensors tested)	In this study, two calibration setups were developed. 1. Chamber setup: In this setup, test sensors and reference instruments were placed inside a chamber of volume approximately 50 L. An aerosol generator associated with a nebulizer was used to generate particulates, and an agitating fan was used to achieve a uniform concentration of particles throughout the chamber. 2. Low-speed duct setup: In contrast to placing sensors and reference instruments inside the chamber as mentioned in setup 1, they were placed in a low air-speed duct system with an exponentially decaying particle concentration. The particulates were injected into the duct from a mixing chamber which is connected to an atomizer and a nebulizer. Grim (model 1.209) was used as a reference instrument in both the setups and particles were generated with a five wt% potassium chloride (KCl) solution through an atomizer.	Ahn et al. [111]
HPMA115S0 (Honeywell)	Sensors and reference instruments were placed inside a test chamber of 125 L constructed using acrylic sheets. The edges were sealed with rubber strips and silicone sealant (a substance used to block the passage of fluids) to prevent leakages. Humidity generators and heat pumps were used to maintain a stable temperature and relative humidity. An aerosol generator was used to generate particulates. Grim (model EDM 107) was used as a reference device.	Omidvarborna et al. [112]
HPMA115S0 (Honeywell), OPC-R1 (Alphasense), SDS018 (Novafitness), SPS030 (Sensirion), and PMS5003 (Plantower)	Sensors were tested inside a 1 m${}^{3}$ chamber made with perspex and stainless steel held within aluminium frames. Internal fans were arranged to mix air inside the chamber, and the temperature was maintained between 25.9 ${}^{\circ }$C and 28.7 ${}^{\circ }$C. A mist generator was used to achieve higher humidity levels up to 95%. Particulates were generated from burning incense sticks and pumped into the chamber via a 5 L/min mass flow controller.	Bulot et al. [113]
PMS A003 (Plantower)	A steel chamber equipped with a sampling inlet, vacuum exhaust and fans was used as a calibration chamber. Test sensors were kept inside the chamber and injected with different concentrations of particulates. The particles were generated by using three methods and injected into the chamber through the sampling inlet. Burning incense sticks, Dispersion of talcum powder and a generation of droplets with collision nebulizer (CH Technologies) using sodium chloride (NaCl) and oleic acid were the three methods of particle generation. Aerodynamic Particle Sizer (APS, TSI Inc., model no. 3321) connected with a scanning mobility particle sizer (SMPS, TSI Inc., model 3082), a pDR-1200 (Thermo Scientific Corp., Waltham, MA, USA), a light-scattering nephelometer with a built-in filter and a Teflon filter were used as reference methods/instruments.	Zamora et al. [106]
PMS 1003 and PMS 3003 (Plantower)	Laboratory calibration was performed in a low-speed wind tunnel operated at a wind speed of 0.5 m/s. Particles were generated using a dry-dust generator (SAG 410, Topas Gmbh, Dresden, Germany). The generated particles were injected into the tunnel with the help of a particle dispersion system that had a nozzle projected into the tunnel. A motor was used to move the nozzle back and forth to disperse the particles throughout the wind tunnel. GRIMM (model 1.109) and TSI DustTrack (model 8530) were used as reference instruments.	Kelly et al. [114]

**Table 6:** Various laboratory calibration setups for gas sensors.

6a. O_3_ Sensors			
**Sensors Tested (Manufacturer)**	**Sensor Type**	**Calibration Setup Details**	**Authors**
OX-B421 (AlphaSense)	Electrochemical	At first, the O_3_ sensors were initially zeroed by using zero gas to test the offset. Then the sensors were calibrated by using Environics S6100, a certified multi-gas calibrator that had an internal O_3_ generator. The sensors were tested in the range of 10 to 1000 ppb concentration levels. Thermo Environmental Instruments (TEI) 49C UV absorption ozone analyser was used as a reference instrument certified by USEPA.	Pang et al. [115]
OX-B421 (AlphaSense)	Electro chemical	Sensors are tested inside a chamber. They obtained CO and NO concentrations by diluting standard gas with zero air. Dynamic dilution calibrator (T700U, Teledyne-API) and standard NO gas are used to produce NO_2_ and O_3_. A computer-controlled flow rate is maintained throughout the experiment. It is Tested for linearity, selectivity, and initial bias without pollutants	Wei et al. [85]
O_3_ Sens 3000 (Unitec), NanoEnvi (Ingenieros Assessores), MiCS 2610 (SGX Sensortech), SP-61 (FIS)	Metal oxide	Sensors placed inside an “O” shaped chamber. MicroCal 5000 gas generator is used for O_3_ production. Interfering gasses are produced with a Self designed Permeation system (for Ammonia (NH_3_), SO_2_, NO_2_) and permeation tubes (for Nitric acid (HNO_3_)) from other manufacturers. LabVIE software is used to control the chamber conditions.	Spinelle et al. [116]
AQMesh	na	Sensor placed in a chamber made up of borosilicate glass. Temperature and relative humidity were maintained as constant throughout the experiment at 20 °C and 30% respectively. Gaseous concentrations obtained by using standard dilution setup. Dilution system details are not available.	Castell et al. [117]
MiCS-4514 (SGX sensortech)	Metal oxide	Test sensors were placed inside a chamber, and a calibrated O_3_ gas was injected into the chamber. The O_3_ gas was generated by using a 2B Technologies™ ozone calibration device (model 306), and a 2B Technologies™ ozone monitor (model 106-L) was used as a reference instrument. Sensors were tested in the temperature range of 13.8 °C to 40.8 °C. The low temperature was obtained using the Danby freezer (model DCFM050C1), and the high temperature was achieved using a seedling heating mat (NAMOTEK 120 V). Relative humidity was adjusted with the help of an ultrasonic atomizer.	Sayahi et al. [118]
S300 with OZU sensor (Aeroqual)	Metal oxide	Sensors mounted on a rack inside a Perspex box that had the facility to draw filtered ambient air. A string fan was used inside the chamber to mix the air. O_3_ was generated inside the chamber by using a controllable, shielded UV source. The sensor’s resistance was calibrated for different concentrations of O_3_.	Bart et al. [119]
OX-B421 (AlphaSense)	Electro chemical	A 3D printed PLA flow cell was used as a calibration chamber, and sensors were housed inside the chamber. The chamber was connected with stainless steel gas lines through which test gasses were injected. At first, sensors were tested for zero reading by pumping zero air and then tested for different standard O_3_ gas concentrations. A gas dilution device and a mercury UV lamp were used to generate different Different O_3_ concentrations, and Thermo Environmental Instruments (TEI, model 49C UV absorption analyser was used as a reference instrument.	Lewis et al. [64]
**6b. NO_2_ Sensors**			
NO_2_-B43F (Alphasense)	Electro chemical	Sensors are placed in an aluminium container. Calibration chamber is connected with two gas paths. One for ambient air another for NO_2_ free air for zero gas calibration. Tested temperature and humidity effects on sensors to correct long term drift due to those effects	Sun et al. [98]
NO_2_-B42F (AlphaSense)	Electro chemical	Sensors are tested inside a chamber. They obtained CO and NO concentrations by diluting standard gas with zero air. Dynamic dilution calibrator (T700U, Teledyne-API) and standard NO gas are used to produce NO_2_ and O_3_. A computer-controlled flow rate is maintained throughout the experiment. It is Tested for linearity, selectivity, and initial bias without pollutants	Wei et al. [85]
NO_2_-A1 (AlphaSense)	Electro chemical	Sensors were kept inside a chamber made with perspex sheets, and a calibrated NO_2_ gas was fed into the chamber. Calibrated NO_2_ gas was obtained by mixing a 9.94 ppm (±2%) NO_2_ standard gas with zero air. Zero air was generated by passing particles filtered ambient air through Whatman zero air generator (Model 76-818, USA). Thermo Environmental Model 42C NO-NO_2_-NO_x_ analyser was used as a reference instrument.	Mead et al. [62]
Not available (AlphaSense)	Electro chemical	Sensors were placed in a chamber and tested for zero reading. The zero reading was tested by pumping the pure air (zero air) into the chamber. Once the zero testing was done, the sensors were calibrated for test NO_2_ gas concentrations. However, the calibration chamber details and how the test gas was produced are not available in the study. The reference grade instrument used in this study was 2B Technologies NO_2_ Monitor (Model 410/401)	Jerrett et al. [120]
**6c. CO SENSORS**			
MiCS-5525 (SGX Sensortech)	Metal oxide	Sensors were placed in an Aluminium enclosure. Mixing manifold fed with dry air, humid air and standard CO gas was used to produce different concentrations of CO. Duty cycled lamp was used to maintain a stable temperature inside the chamber. Temperature and flow rate were controlled with LabVIEW software.	Masson et al. [59]
CO-B4 (AlphaSense)	Electro chemical	Sensors are tested inside a chamber. They obtained CO and NO concentrations by diluting standard gas with zero air. Dynamic dilution calibrator (T700U, Teledyne-API) and standard NO gas are used to produce NO_2_ and O_3_. A computer-controlled flow rate is maintained throughout the experiment. It is Tested for linearity, selectivity, and initial bias without pollutants	Wei et al. [85]
Not available (AlphaSense)	Electro chemical	Sensors were placed in a chamber and tested for zero reading. The zero reading was tested by pumping the pure air (zero air) into the chamber. Once the zero testing was done, the sensors were calibrated for test NO_2_ gas concentrations. However, the calibration chamber details and how the test gas was produced are not available in the study. The reference grade instrument used in this study was TSI Q-trak (model 7565)	Jerrett et al. [120]
MiCS- 5525 (SGX Sensortech)	Metal oxide	A Teflon-coated aluminium chamber connected with mass flow controllers (Coastal Instruments FC-2902V) was used to calibrate sensors. The chamber was equipped with temperature and humidity control mechanisms. Instead of placing sensors (the number of sensors tested was 13) directly inside the chamber, they were first placed in a steel carousel type enclosure to maintain uniform gas distribution to all the sensors. A certified CO gas was injected into the chamber, and solenoidal valves were used to control the flow rate. LabVIEW software (LabVIEW 2011) and Labjack data acquisition devices (LabJack U3-LV) were used for instrument control and data logging. Temperature variations were controlled with the help of a heat lamp.	Piedrahita et al. [16]

**Table 7:** In field calibration models tested with various sensors at different locations. Here, T is Temperature, RH is relative humidity, AH is absolute humidity, cal* is calibration.

Calibration Methods Tested	Pollutants	Senors Used (Manufacturer)	Parameters Considered for Calibration	Location (Country)	Authors, Year
1. Single variable linear regression 2. Polynomial multiple variable regression	PM_2.5_	PMS5003 (Plantower)	PM_2.5_, RH	Athens, Ioannina (Greece)	Stavroulas et al., 2020 [99]
1. Multisensor data fusion with weighted averages 2. multisensor data fusion with machine learning	O_3_	OX-B431 (AlphaSense), MICS-2614 (Sensortech)	O_3_, T, RH	Several locations (Spain, Austria, Italy)	Ferrer-Cid et al., 2020 [93]
1. Two separate linear fits based on threshold for PurpleAir 2. Quadratic multiple regression for Met-one NPM	PM_2.5_	PurpleAir-PA-IIMet-one-NPM	PM_2.5_, T, RH	Four locations (USA).	Malings et al., 2020 [124]
Multiple regression with kriging estimation correction	O_3_	MICS-2614 (Sensortech)	O_3_, T, RH	Spain, Austria, Italy	Barcelo et al., 2019 [24]
Multiple regression with iterative bayesian approach	NO_2_	NO_2_-3E50 (Citytech Sensoric)	NO_2_, T, RH O_3_, wind speed, wind direction	Several locations (Netherlands)	Zoest et al., 2019 [94]
1. lienar regression 2. Multiple regression 3. ANN with different training methods	CO O_3_	CO-B4, NO_2_-B4 (Alphasense)	for CO cal* CO, PM_2.5_, NO_2_ for O_3_ cal* O_3_, NO, AH	Stari Grad (Serbia)	Topalović at al., 2019 [100]
1. liner regression, 2. multiple regression, 3. eXtreme Gradient Boosting 4. Feed forward neural networks	PM_2.5_	PMS50003 (Plantower)	PM_2.5_, T, RH	Calgary Region (Canada)	Minxing et al., 2019 [131]
1. Multiple regression 2. GAM generalized additive models	NO_2_ NO	Emotes containing AlphaSense sensors	Mean values of NO_2_, NO, RH, T, wind speed	Sheffield (UK)	Munir et al., 2019 [95]
K-nearest neighbours	SO_2_	SO_2_-B4 (AlphaSense),	SO_2_, RH, T	Hawai‘i (USA)	Hagan et al., 2018 [101]
1. Multiple regression 2. multiple regression with regularization 3. Gradient boosting regression tree model	PM_2.5_	PPD42 (Shinyei)	PM_2.5_,T, RH, barometric pressure, precipitation, dew point	New York (USA)	Johnson et al., 2018 [84]
1. liner fit with temp and RH correction 2. quadratic fit with temp and RH correction	PM_2.5_	Plantower PMS3003,	PM_2.5_, T, RH	Kanpur (India), Durham (UK)	Zheng et al., 2018 [133]
Multiple regression combined with machine learning models of SVM, RF and scaled ANN different model combinations at different concentrations	NO_2_	AQmesh pods	NO_2_, NO, O_3_, T	Madrid (Spain)	Cordero et al., 2018 [96]
High dimensional model representation (HDMR)	NO, CO	NO-B4, CO-B4 (AlphaSense),	for NO cal* NO, temp for CO cal* CO, temp	Cambridge (UK)	Cross et al., 2017 [23]

**Table 8:** Post-deployment calibration strategies.

Calibration Method	Previous Studies
Blind calibration: In blind calibration, sensors are calibrated to the nearby reference stations when it is believed that both the sensors and the reference stations are exposed to the same concentrations. Advantages: Simple Can calibrate both stationary and mobile sensors Limitations: Has to wait until certain condition is reached like concentration is below certain level. Possible to calibrate only gain and offset	Jiao et al., calibrated a network of sensors against the nearby reference stations between 01.00 A.M. and 4.00 A.M. by assuming similar atmospheric conditions throughout the monitoring site [79]. Moltchanov et al., also followed the same procedure as Jiao et al., to calibrate O_3_ and NO_2_ sensors deployed in Haifa city by assuming minor spatial variations of concentrations of O_3_ and NO_2_ during 1.00 A.M. and 4.00 A.M. throughout the experimental area [27]. Broday et al., also followed the same procedure as Jiao et al., to calibrate O_3_ sensors after post-deployment [142] Tsujita et al., calibrated offset of NO_2_ sensors deployed in Tokyo city to the reference stations when they show the same concentration below 10 ppb [143]. Muller et al., calibrated O_3_ and NO_2_ based on the assumption that inner-city concentrations are lower at night and outer city concentrations are higher in the afternoon. Therefore, they calibrated O_3_ and NO_2_ sensors deployed in the inner city to the reference stations present at the same locality at night, and sensors present at the outer city were calibrated to the available reference stations in that locality in the afternoon [144].
Collaborative calibration: In collaborative calibration, a mobile sensor is calibrated to a reference station when they meet in space and time, and it is called as sensor rendezvous with a reference station. Advantages: Able to calibrate mobile sensors Possibility of better calibration accuracy when compared to other methods. Limitations: Able to calibrate mobile sensors Possibility of better calibration accuracy when compared to other methods.	Saukh et al., used Collaborative calibration to calibrate OpenSense devices (sensor devices made with LCS) mounted on streetcars to monitor air quality in an urban area. Total ten sensor devices were placed on 10 streetcars, and they were calibrated to two reference stations present in the monitoring path. Ordinary least squares was used as a calibration method [145] Hasenfratz et al., used a collaborative calibration technique to calibrate low-cost gas sensors mounted on public transport vehicles. When the vehicles encountered the reference stations during the flight, they collected the reference station data and later, it was used to calibrate the sensors to compensate the drift error [146] Miluzzo et al., proposed CaliBree, a collaborative self-calibration system for sensors carried by a human moving at a walking speed. Reference stations available in the nearby vicinity were used to calibrate the sensors. When the sensors were rendezvous with the reference stations, the CaliBree automatically calibrated the sensors to the corresponding accurate measurements [147]
Multi-hop calibration: Multi-hop calibration extends the collaborative calibration. In Multi-hop calibration a freshly calibrated sensor instead of reference station/instrument is used to calibrate another sensor when they meet in space and time. Then the calibrated sensor is used to calibrate another sensor and the chain continues until the calibration finished for all the sensors. Advantages: No need of reference station/instrument everywhere in the measurement process. Suitable for mobile sensor monitoring Limitations Sensors are used to calibrate other sensors instead of reference instrument/station that causes error accumulation. Hence, sensors at the end of the chain are more prone to wrong calibration. Linear calibration models are not suitable due to error accumulation problem.	Xiang et al., used multi-hop calibration technique to eliminate the manual calibration of sensors which takes more time and effort. They considered the data of nearby sensors to calibrate other sensors in the network. A specially designed algorithm addressed the error accumulation problem over the chain in multi-hop calibration [148] Saukh et al., proposed GMR (geometric mean regression) to calibrate the sensor devices placed on vehicles. Ten sensor devices were placed on ten different vehicles, and two reference stations available in the monitoring path were used for the initial calibration purpose. Once the initial calibration with the reference station was finished, then the freshly calibrated sensor devices were used to calibrate other sensor devices. However, every sensor rendezvous was not considered for calibration purposes. Rendezvous, in which the correlation between two sensor readings was more than 0.5, was considered as a valid rendezvous point. A distance of 50 m and a duration of 5 min were considered as the rendezvous characteristics between two sensor devices, and one stretch of measurement was continued for ten days [149] Maag et al., proposed sensor array network calibration (SCAN), a multi-hop calibration technique to calibrate the sensor arrays mounted on streetcars to monitor air pollution in the city of Zurich, Switzerland. A total of 11 sensor arrays were placed on 11 different streetcars, and each sensor array was considered as one hop. In the calibration process, at first, they calibrated a hop with the reference station when it was rendezvous with the reference station. Then, the calibrated hop was used to calibrate other hops in the network when it was rendezvous with other hops. A distance of 50 m between hops and a time span of 5 min with 200 samples was considered as rendezvous parameters. The proposed calibration method SCAN was able to address the error accumulation problem in the multi-hop calibration technique [134] Fu et al., introduced a new method, K-hop calibratability, a multi-hop calibration technique to calibrate the sensor devices placed on city busses with the help of other calibrated sensors. At the same time, they introduced the optimal placement of reference devices in the sensor’s rendezvous path so that each sensor was k-hop calibratable [150]
Transfer calibration: Calibration transfer can be done by transferring the calibration parameters of a source sensor to a target sensor. Here the target sensor is the sensor of interest to calibrate and the source sensors is the one which is having access to the reference station. At first the source sensor is calibrated to the reference station then the calibration parameters are transferred to the target sensors based on some learning theory. Advantages: Both stationary and mobile sensors can be calibrated. Limitations Need identical sensors;	Basically transfer calibration is used for the mass calibration of electronic noses, odour detection devices used in chemical industries [126,127,151]; Fonollosa et al., calibrated eight metal oxide gas sensors using transfer calibration. In the calibration process, one out of all the sensors was selected as a master sensor, and the rest were considered as slave sensors. Then the outputs of slave sensors were standardized to the master sensor’s output, and the master sensor was calibrated against the reference instrument. Once the standardization was finished, the calibration parameters of the master sensor were directly transferred to slave sensors. The whole process can be represented in equation *S_master_* = *S_slave_F*. Here, *S_master_* is the output of the master and *S_slave_* is the out of the slave [152]; Recently, Cheng et al., proposed ICT (Infield calibration transfer) to calibrate network of particulate sensors deployed in Beijing city, China. In stead of direct transfer of the calibration parameters, ICT works on statistical calibration transfer, which makes use of the similarity in distributions at the source site (where the source sensor measures) and target site (where the target sensor measures) [125].

## Data Availability

Not applicable since this article is a review article, and we are not using any data that we generated.

## Supplementary Materials

The following are available at https://www.mdpi.com/article/10.3390/s1010000/s1, Figure S1: Graphical representation of minimizing available LCS set with our procedure (Algorithm S1 in Supplementary Materials), that makes the selection of sensors is easy from a minimal set. Table S1: Evaluation metrics for sensor vs reference instrument comparison. Table S2: Metric to test reproducibility and repeatability of LCS for AQM. Algorithm S1: Procedure to select LCS.
