# Supplementary material for: Establishing A Sustainable Low-Cost Air Quality Monitoring Setup: A Survey of the State-of-the-Art
Source: Sensors (Basel). 2022 Jan 5;22(1):394. doi: 10.3390/s22010394 (PMC8749853; doi:10.3390/s22010394)
Supplement: Supplementary file 1 [file sensors-22-00394-s001.zip › sensors-1488737-supplementary.pdf]

MDPI

Article

Establishing a sustainable low-cost air quality monitoring setup: a survey of state of art

M. V. Narayana [1]*, Devendra Jalihal [1] and Shiva Nagendra S.M. [2]

[1] Electrical Engineering, Indian Institute of Technology, Madras
[2] Civil Engineering, Indian Institute of Technology, Madras
* Correspondence: ee18d302@smail.iitm.ac.in

Version December 9, 2021 submitted to Journal Not Specified

supplements

Table S1. Evaluation metrics for Sensor vs reference instrument comparison. In this table s_i is sensor value and r_i is reference grade instrument value and $\bar{s}$, $\bar{r}$ are their respective means. n is number of observation N is total number of observations. d is the difference in the paired ranks of s and r values

metric	equation	inferences
Correlation coefficient (r)[1,2]	$r = \frac{\sum(s_i-\bar{s})(r_i-\bar{r})}{\sqrt{\sum(s_i-\bar{s})^2\sum(r_i-\bar{r})^2}}$	Indicates linear relationship between sensor measured values and corresponding reference instrument values
Spearman's correlation coefficient (ρ) [3]	$\rho = \frac{6\sum {d_i}^2}{n(n^2-1)}$	Indicates the monotonic relationship between sensor measured values and reference corresponding reference instrument values
Mean Absolute error (MAE) [4]	$MAE = \frac{1}{N}\sum_{i=1}^{i=n}\lvert s_i - r_i\rvert$	Indicates the average difference between the sensor values and reference instrument values
Mean bias error (MBE) [5]	$MBE = \frac{1}{N}\sum_{i=1}^{i=n}(s_i - r_i)$	Indicates average bias between the sensor values and reference instrument values
Root mean squared error and (RMSE) Normalized root mean squared error(nRMSE) [6–9]	$RMSE = \sqrt{\frac{1}{N}\sum_{i=1}^{i=n}(s_i - r_i)^2}$ $nRMSE = \frac{\sqrt{\frac{1}{n}\sum_{i=1}^{i=n}(s_i-r_i)^2}}{\frac{1}{2N}\sum_{i=1}^{i=n}(s_i+r_i)}$	It is a good measure of sensor accuracy. Indicates the sensors values dispersion from the refrance grade instrument

Table S2. metric for reproducibility and repeatability. In this table σ is standard deviation and μ is mean

metric	equation	inferences
coefficient of variation (CV) [10]	$\frac{\sigma}{\mu}$	Indicate the precision of sensors

In the figure S1, The leftmost circle represents, all commercial sensors available in the market and rightmost circle represents the final list of sensors after our methodology, out of which we can select the desired. The size of the circle decrease as we progress form left to right since the right one is the subset of the left one. Decreasing size of circle from left to right illustrates decrease in number of sensors in the list. Therefore selecting sensors from left circle is more cumbersome than from the right one.

Algorithm S1 The proposed procedure to select sensors is illustrated in the following steps, and how this procedure can help to reduce the efforts in the selection of sensors is illustrated in figure s1.

Step 1: List the application details such as
Location of the study, duration of the study, availability of reference station near by, meteorology of the site (temperature and humidity), major pollution sources
Step 2: Study the basic statics of the data that is collected in the location i.e.,
Range and mean of temperature & humidity, Wind speed, wind direction and distribution of the pollutants of interest (optional and possible only if near by reference station is available)
Step 3: Find out sensors of which type (MOS/EC/PM/others) suitable in the application.
Every sensor type has certain limitations and advantages [11,12]. Map these details with the data gathered in steps 1 and 2 to figure out suitable sensor type.
Step 4: List of sensors finalized type for the pollutant of interest that are available in the market.
The extensive list reported in the studies [12–16] can help in this regard
Step 5: Study the data-sheets of listed sensors and discard which are not suitable for the study.
This can be done based on characteristics of the sensors mentioned in the data-sheets, like the range of measurement, accuracy, sensitivity. Studies by Maag et al. [17] and Rai et al. [12] useful to understand these characteristics.
Step 6: Check for the peer-reviewed evaluation studies of the remaining sensors in the literature.
According to them, we can further reduce the list of sensors, makes the selection task easy.
Step 7: Do cost trade off
Finally, we can select the best possible LCS suitable for our application.
Step 8: Basic input, output testing to check working of sensors
Step 9: Proceed with calibration and evaluation discussed in the next sections.

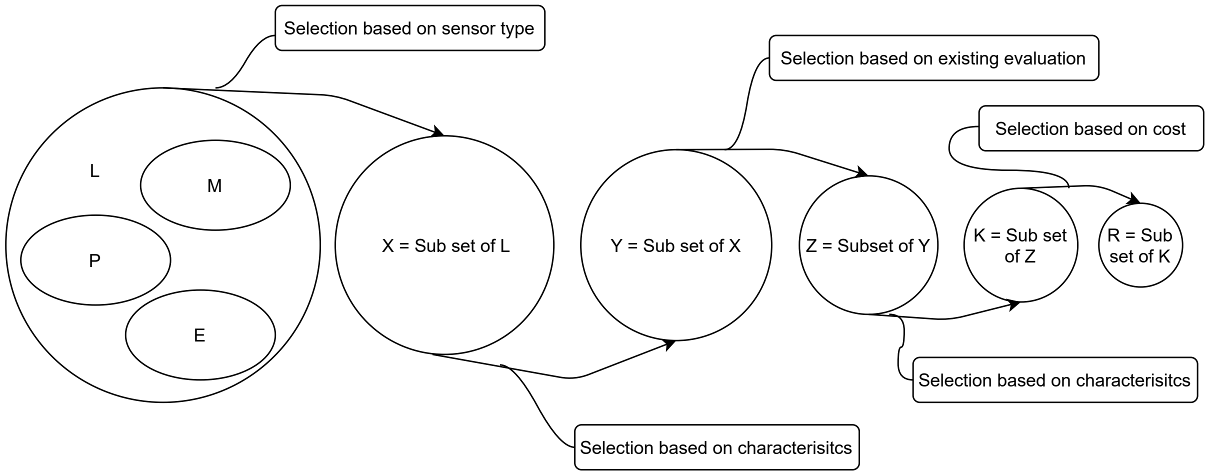

Figure S1. Graphical representation of minimizing available LCS set with our procedure, that makes the selection is easy from a minimal set. Here L : set of all commercial available sensors, M : metal oxide sensors, P : particulate sensors, E : electrochemical sensors, O : L - (M ∪ P ∪ E) represents other sensors of advanced sensing principles like graphene etc. X, Y, Z, K, R are different subsets of L, where R is having minimum number of sensors. Circle size decrements from left to right indicates the reduction of sensor list out of which suitable can select

References

1. Jerrett, M.; Donaire-Gonzalez, D.; Popoola, O.; Jones, R.; Cohen, R.C.; Almanza, E.; de Nazelle, A.; Mead, I.; Carrasco-Turigas, G.; Cole-Hunter, T.; et al. Validating novel air pollution sensors to improve exposure estimates for epidemiological analyses and citizen science. *Environ. Res.* **2017**, *158*, 286–294.
2. Ferrer-Cid, P.; Barcelo-Ordinas, J.M.; Garcia-Vidal, J.; Ripoll, A.; Viana, M. Multisensor Data Fusion Calibration in IoT Air Pollution Platforms.*IEEE Internet Things J.* **2020**, *7*.
3. Jiao, W.; Hagler, G.; Williams, R.; Sharpe, R.; Brown, R.; Garver, D.; Judge, R.; Caudill, M.; Rickard, J.; Davis, M.; et al. Community Air Sensor Network (CAIRSENSE) project: Evaluation of low-cost sensor performance in a suburban environment in the southeastern United States. *Atmos. Meas. Tech.* **2016**, *9*, 5281–5292.
4. Bigi, A.; Mueller, M.; Grange, S.K.; Ghermandi, G.; Hueglin, C. Performance of NO, NO2 low cost sensors and three calibration approaches within a real world application. *Atmos. Meas. Tech.* **2018**, *11*, 3717–3735.
5. Stavroulas, I.; Grivas, G.; Michalopoulos, P.; Liakakou, E.; Bougiatioti, A.; Kalkavouras, P.; Fameli, K.M.; Hatzianastassiou, N.; Mihalopoulos, N.; Gerasopoulos, E. Field evaluation of low-cost PM sensors (Purple Air PA-II) Under variable urban air quality conditions, in Greece.*Atmosphere (Basel).* **2020**, *11*.
6. van Zoest, V.; Osei, F.B.; Stein, A.; Hoek, G. Calibration of low-cost NO2 sensors in an urban air quality network.*Atmos. Environ.* **2019**, *210*, 66–75.
7. Si, M.; Xiong, Y.; Du, S.; Du, K. Evaluation and Calibration of a Low-cost Particle Sensor in Ambient Conditions Using Machine Learning Technologies.*Atmos. Meas. Tech.* **2019**, 1–25.
8. Martin, C.R.; Zeng, N.; Karion, A.; Dickerson, R.R.; Ren, X.; Turpie, B.N.; Weber, K.J. Evaluation and environmental correction of ambient CO2 measurements from a low-cost NDIR sensor. *Atmos. Meas. Tech.* **2017**, *10*, 2383–2395.
9. Zikova, N.; Hopke, P.K.; Ferro, A.R. Evaluation of new low-cost particle monitors for PM2.5 concentrations measurements. *J. Aerosol Sci.* **2017**, *105*, 24–34.
10. Badura, M.; Batog, P.; Drzeniecka-Osiadacz, A.; Modzel, P. Evaluation of low-cost sensors for ambient PM2.5 monitoring. *J. Sensors* **2018** 5096540.
11. J. E. Thompson. Crowd-sourced air quality studies: A review of the literature & portable sensors. *Trends Environ. Anal. Chem.*, **2016** *11*, 23–34.
12. Rai, A.C.; Kumar, P.; Pilla, F.; Skouloudis, A.N.; Di Sabatino, S.; Ratti, C.; Yasar, A.; Rickerby, D. End-user perspective of low-cost sensors for outdoor air pollution monitoring. *Sci. Total Environ.*, **2017**, 607–608, 691–705.
13. Karagulian, F.; Barbiere, M.; Kotsev, A.; Spinelle, L.; Gerboles, M.; Lagler, F.; Redon, N.; Crunaire, S.; Borowiak, A. Review of the performance of low-cost sensors for air quality monitoring. *Atmosphere (Basel).*, **2019**, *10*.
14. C. Borrego et al. Assessment of air quality microsensors versus reference methods: The EuNetAir joint exercise. *Atmos. Environ.*, **2016**, *147 (2)*, 246–263.
15. C. Borrego et al. Assessment of air quality microsensors versus reference methods: The EuNetAir Joint Exercise – Part II.*Atmos. Environ.*, **2018** *193*, 127–142.
16. M. Aleixandre; M. Gerboles. Review of small commercial sensors for indicative monitoring of ambient gas. *Chem. Eng. Trans.*, **2012**, *30*, 169–174.
17. Maag, B.; Zhou, Z.; Thiele, L. A Survey on Sensor Calibration in Air Pollution Monitoring Deployments. *IEEE Internet of things.*, **2018**, *5* 4857 - 4870.

Publisher's Note: MDPI stays neutral with regard to jurisdictional claims in published maps and institutional affiliations.

© 2022 by the authors. Submitted to *Journal Not Specified* for possible open access publication under the terms and conditions of the Creative Commons Attribution (CC BY) license (http://creativecommons.org/licenses/by/4.0/).
